# Seasonal sea ice characterized the glacial Arctic-Atlantic gateway over the past 750,000 years

**DOI:** 10.1126/sciadv.adu7681

**Published:** 2025-07-04

**Authors:** Jochen Knies, Lukas Smik, Pengyang Song, Monica Winsborrow, Henning A. Bauch, Gerrit Lohmann, Simon T. Belt

**Affiliations:** ^1^ Geological Survey of Norway, Trondheim, Norway.; ^2^ iC3: Centre for ice, Cryosphere, Carbon and Climate, Department of Geosciences, UiT The Arctic University of Norway, Tromsø, Norway.; ^3^ Biogeochemistry Research Centre, School of Geography, Earth and Environmental Sciences, Plymouth University, Plymouth, UK.; ^4^ Centre for Resilience in Environment, Water and Waste (CREWW), College of Life and Environmental Sciences, University of Exeter, Exeter, UK.; ^5^ Alfred Wegener Institute, Helmholtz Centre for Polar and Marine Research (AWI), Bremerhaven, Germany.; ^6^ GEOMAR Helmholtz Centre for Ocean Research, Kiel, Germany.; ^7^ MARUM–Centre for Marine Environmental Sciences, University of Bremen, Bremen, Germany.

## Abstract

The past occurrence of an extreme ~1-kilometer–thick Arctic Ocean–Nordic Seas ice shelf has been inferred from submarine landscape features and geochemical records, although fundamental aspects of its characteristics, impacts, and timing remain highly debated. Here, we challenge this pan-Arctic glaciation hypothesis by investigating two sites from the Arctic-Atlantic gateway (AAG) and the Nordic Seas. Suborbital to millennial-scale surface water bioproductivity changes provide no evidence for a continuous ice shelf in the AAG and the Nordic Seas over the past ~750,000 years. Instead, proxy data and model simulations reveal the persistent presence of seasonal sea ice cover and open water phytoplankton blooms during both glacial and interglacial times. If the AAG and Nordic Seas were ever covered by an ice shelf during these times, then it must have been a partial, or at best, a very short-lived glacial phenomenon.

## Teaser

The glacial Arctic-Atlantic gateway was covered by sea ice after the Mid Pleistocene transition, not a grounding ice shelf.

## INTRODUCTION

**Fig. 1.**
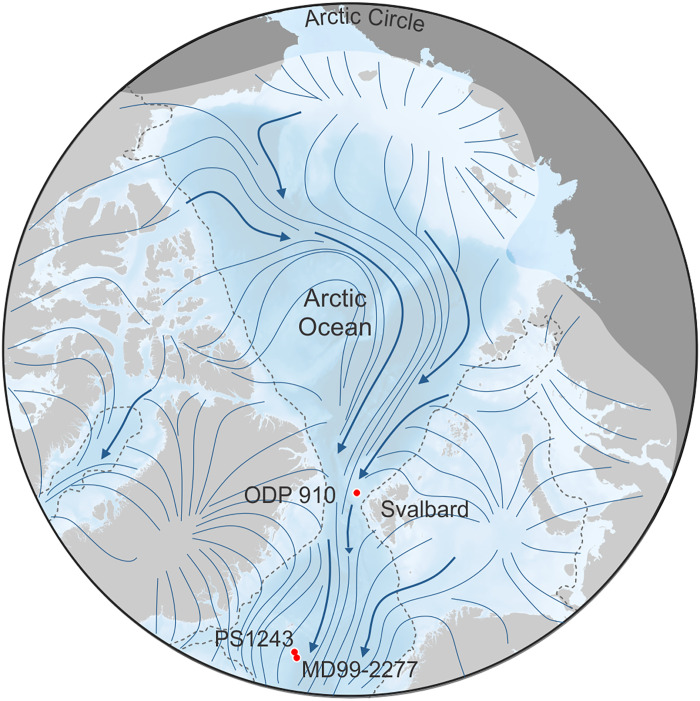
Site locations and hypothesized pan-Arctic glaciation. ODP Site 910 and MD99-2277 (red dots) are located in the Eurasian sector of the Arctic Ocean and central Nordic Seas, respectively. Site 910 lies just beyond the margins of the maximum MIS 2 Eurasian ice sheets [gray dashed line; based on (*51*)], while MD99-2277 was simultaneously covered by sea ice. However, both sites are hypothesized to have been fully glaciated under the maximum pan-Arctic ice sheet extent (white shaded extent with blue flow lines) proposed by Hughes *et al.* (*75*) [modified from Jakobsson *et al.* (*7*) and Geibert *et al.* (*3*)].

The rapid loss of Arctic sea ice over the last 30 years resembles boundary conditions that have been reconstructed for the last extreme warmth, the mid-Pliocene warm period, ~3 million years ago (Ma ago) (*1*, *2*). In Earth System models, this “warm” end-member of the Arctic Ocean contrasts with “cold” Arctic climate states when extensive ice sheets covered circum-Arctic landmasses during the Pleistocene ice ages. A key uncertainty of this “cold” end-member is whether it ever included a continuous Arctic Ocean–Nordic Seas ice shelf (up to 1-km thick) (Fig. 1) (*3*). Support for such a pan-Arctic glaciation, proposed by Mercer (*4*), has come from interpretation of seafloor landforms (*5*–*8*) and marine sediments (*3*); however, constraining the age (both absolute and relative) of these remains highly problematic (*9*–*11*). To narrow uncertainty around key characteristics of the extreme “cold” Arctic climate state, new, targeted, and well-dated proxy records are required.

Here, we study “cold” Arctic climate states following the middle Pleistocene transition (MPT, ~1.2 to 0.7 Ma) until the Last Glacial Maximum (LGM) by integrating new bioproductivity records from two sites from the Arctic-Atlantic gateway (AAG) and the Nordic Seas (Fig. 1) with simulations using the AWI Earth System Model (AWI-ESM2), a fully coupled model with ocean, atmosphere, and dynamic vegetation components, and prescribed glacial boundary conditions including ice sheet extent and orbital forcing (*12*, *13*), for extreme glacial Marine Isotope Stage (MIS) 6 and LGM climate states (see the Supplementary Materials for details). Our bioproductivity records are based on source-specific and seasonally sensitive lipid biomarker proxies. Specifically, we use the highly branched isoprenoid (HBI) IP_25_ as a marker for seasonal sea ice owing to its production by certain Arctic sympagic (sea ice–associated) diatoms (*14*, *15*) during spring and deposition in underlying sediment following release from melting ice in late spring-early summer (*16*). To complement the IP_25_ data, we measured epi-brassicasterol and dinosterol, two sterols that are diagnostic of open-water (i.e., pelagic) plankton productivity during spring (diatoms) and summer (dinoflagellates), respectively (*17*, *18*). Collectively, this suite of organic geochemical proxies provides evidence for seasonal sea ice (i.e. autumn-late winter) and the transition to open water conditions (late spring-summer) and was applied to Ocean Drilling Program (ODP) Hole 910A (hereafter referred to as 910A) on the Yermak Plateau, NW Svalbard. Further, x-ray fluorescence (XRF) calcium records are used as a proxy for surface water production of calcareous planktic foraminifera derived from piston core MD99-2277 in the central Nordic Seas (see the Supplementary Materials). Based on the continuous presence of these surface water bioproductivity indicators in the AAG and Nordic Seas, with a time resolution of ~1000 to ~6500 years per sample over the past 750,000 years, we conclude that if a pan-Arctic ice shelf expanded to these regions during this period, it must have been short-lived, building-up and collapsing within ~6000 to 7000 years.

## RESULTS

### Arctic sediment age constraints

**Fig. 2.**
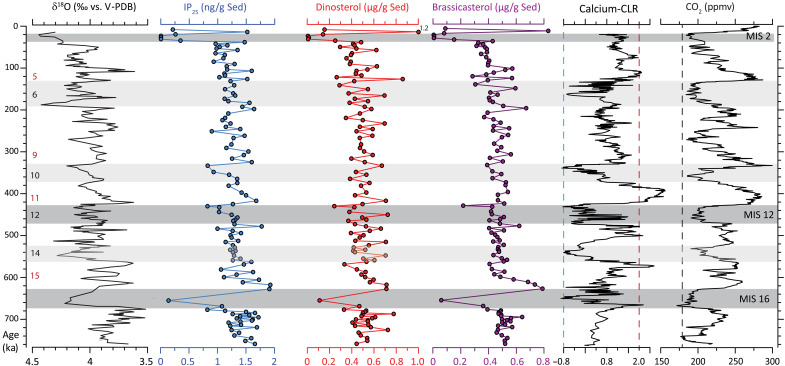
Pleistocene climate proxy data. Planktonic δ^18^O (‰), and concentrations of IP_25_ (in ng/g Sed), and brassicasterol and dinosterol (in μg/g Sed.) from 910A, and XRF calcium (centre log ratio, clr) from MD99-2277 against age (ka = *1000 years). Antarctic *p*CO_2_ record from (*55*). Glacial and interglacial MISs are indicated by black and red numbers, respectively, and vertical stippled lines indicate XRF-Ca (clr) derived boundaries for high (red = 2.0) and low/zero (blue = −0.8) bioproductivity (see the ‘Arctic sea ice since Mid-Pleistocene’ section). Black vertical stippled line indicates lowest CO_2_ value during the Quaternary (<180 ppmv).

The chronologies for our 750,000-year long bioproductivity records have been published elsewhere (*19*–*21*); however, given that chronostratigraphic challenges are common hinderances to robust Arctic proxy reconstructions, we here summarize their key elements and provide further details in the Supplementary Materials. The stratigraphic framework for 910A and MD99-2277 [the latter includes nearby core PS1243 (69.3718°N, 6.553°W, 2711-m water depth)] are derived from continuous stable isotope records of polar planktic foraminifera (*Neogloboquadrina pachyderma*) combined with paleomagnetic vectors and biostratigraphic datum (*19*–*21*). The highest occurrence of the calcareous nannofossil *Pseudoemiliania lacunosa* at 10.45 m below sea floor (mbsf) and AMS^14^C datings at 0.35 mbsf constrain the upper sequence of 910A to between ~440,000 (late MIS 12) and 20,000 years (MIS 2) ago. Glacial maxima between MIS 12 and MIS 2 are identified on the basis of the heaviest planktic δ^18^O values (>4‰) (Fig. 2), a widely accepted approach to identify glacial maxima in high northern latitudes (*22*). Toward the base of 910A, the placement of the Brunhes/Matyuama (B/M) magnetic boundary (~0.78 Ma) at 19.45 mbsf is key for assigning older glacials beyond MIS 12. For MD99-2277, the B/M boundary was identified at 18.84 m (*20*). Alternating glacial-interglacial cycles are constrained by aligning high-resolution stable isotopes and other records with well-established age models of core PS1243, which extends to MIS 11 (*20*, *21*, *23*). Beyond MIS 11, ages are assigned by correlating the planktic δ^18^O and inclination records to global δ^18^O records (*24*–*26*). The sedimentation rates at 910A and MD99-2277 vary between 0.8 and 9.6 cm/1000 years, resulting in a mean resolution of ~1000 years (MD99-2277) and ~6500 years (910A) per sample.

### Arctic sea ice since Mid-Pleistocene

We identified the spring sea ice biomarker proxy IP_25_ in all 910A samples, indicative of seasonal sea ice with late spring–early summer open-water conditions. The lowest concentrations occur during the LGM and MIS 16 (~676,000 to 621,000 years ago) (Fig. 2). Generally, IP_25_ concentrations follow the middle to late Pleistocene glacial-interglacial cycles in showing high and low values during warm and cold periods, respectively. Further evidence for late spring–summer open-water conditions is evident from the continuous occurrence of the pelagic phytoplankton biomarkers epi-brassicasterol and dinosterol in 910A, with most prominent lows during the LGM and MIS 16, as per IP_25_ (Fig. 2). The XRF-Ca (clr) record reflects the abundance of planktic foraminifera in MD99-2277 and is used as a proxy for bioproductivity changes in central Nordic Seas surface waters (*27*). Clearly, the highest bioproductivity of calcareous zooplankton in surface waters occurred during interglacials MIS 5e, 11, 13, 15, and potentially 17 [taking into account a placement error of the MIS 16/17 boundary in the age model (*20*, *21*)] with XRF-Ca (clr) > 2. Three short intervals (~1000 to 2000 years) with XRF-Ca clr lower than −0.8 (corresponding to a zero number of planktic foraminifera) occur during glacial MIS 10, 12, and 16, at the same time as low IP_25_ concentration is recorded in 910A (Fig. 2).

Based on the continuous presence of IP_25_, dinosterol and epi-brassicasterol in 910A, we find no evidence that the eastern AAG has been influenced by a floating/grounded ice shelf during the past ~750,000 years on suborbital timescales, consistent with earlier interpretations of glacial landforms on the Yermak Plateau crest, being the result of drifting megabergs from the Arctic interior and not of a major grounded ice sheet (*28*, *29*). These icebergs, with drafts exceeding 800 m, must have had prolonged residence times in the Arctic Ocean during past glaciations. At times, these large icebergs became grounded in shallower regions or became immobilized along continental shelf edges. Their probable sources include the northern margins of Greenland, the Canadian Arctic Archipelago, and the Barents-Kara Seas—Archipelago regions that exhibit well-preserved glacial geomorphological features indicative of former major ice streams. Our data therefore challenge model inferences (*30*, *31*) that grounding of an ice shelf in the central Arctic Ocean (i.e., Lomonosov Ridge and East Siberia) requires an Arctic-wide ice shelf including the Yermak Plateau. We further note that the timing (~5000 to 15,000 years) required to build up an ice shelf and reach sufficient thickness to ground on both the Lomonosov Ridge (>1200 m) and the Yermak Plateau (>700 m) (*6*, *7*, *28*–*30*) is at the lower end of that indicated by the suborbital sampling resolution of our sea ice–pelagic biomarker records in 910A (~6500 years). Our findings for 910A are supported further by millennial-scale low (glacial) and high (interglacial) bioproductivity in MD99-2277 from the central Nordic Seas (Fig. 2). The possible grounding of glacial ice on the Jan Mayen Ridge (~60-km west of MD99-2277) inferred from submarine landforms (*8*) must thus have been partial, extremely short-lived (~1000 to 2000 years) or occurred during glaciations before 750,000 years ago. The clearest evidence—both from borehole and geophysical data—for a possible grounding/floating ice shelf beyond the shelf edge in the AAG region is only evident for the Mid-Pleistocene transition (MPT), sometime between ~950,000 and 790,000 years ago (*19*, *29*, *32*). We exclude the possibility of lateral transport of phyto- and zooplankton by subglacial water masses, not least because a floating ice shelf on the Yermak Plateau (AAG) and the Jan Mayen Ridge (Nordic Seas), with an assumed thickness of ~1-km, would have prevented northward transport of Atlantic-derived water masses reaching the shallow crest (~500 m) of the Yermak Plateau (910A; Fig. 1). Furthermore, the diagenetic overprint of biomarkers is unlikely as their concentration profiles largely follow the amplitude trends in XRF-Ca (clr) (Fig. 2).

The increase in concentration of sea ice and pelagic diatom/dinoflagellate biomarkers followed by XRF Ca (clr) > 2 during prominent interglacial peaks (e.g., MIS 5e, 15, and 11c) points to seasonal sea ice conditions and regular zooplankton blooms in the AAG and Nordic Seas, implying constant influence of warm Atlantic-derived water masses and biological productivity along a Marginal Ice Zone (MIZ). Similarly, lower IP_25_ values during glacial periods (e.g. MIS 2, MIS 10, MIS 12, and MIS 16) are within reported ranges on the Yermak Plateau during MIS 6 (*10*, *33*, *34*) and occur alongside reduced pelagic biomarker concentrations in 910A (Fig. 2). The inferred limited bioproductivity during these glacial periods is likely due to polynyal development driven by katabatic winds from an expansive marine-based ice sheet and sustained by subsurface inflow of Atlantic waters in the eastern Nordic Seas as suggested from previous LGM/late MIS 6 sea ice and productivity studies (*27*, *33*, *35*–*43*). Low XRF-Ca (clr) values during MIS 6, MIS 10, MIS 12, MIS 14, and MIS 16 occur on millennial timescales and indicate short-lived extreme sea ice conditions in the central Nordic Seas in an environment that is generally characterized by massively enhanced input of ice-rafted sediments—effectively causing dilution of calcite bearing plankton deposition—and a variable sea ice coverage largely controlled by the in- and outflow of Atlantic and Arctic waters. Indeed, superimposed on glacial-interglacial fluctuations, distinct millennial-scale climate variability in the elemental Si/Sr ice-rafted debris (IRD) proxy in the open North Atlantic (site U1308) (*44*) corroborates these bioproductivity changes in the AAG and central Nordic Seas during the past 750,000 years, and further supports our inference of a sea ice/iceberg—open water dominated regime in the Nordic Seas rather than the prevalence of a continuous Arctic Ocean–Nordic Seas ice shelf.

### Arctic climate in ESMs

**Fig. 3.**
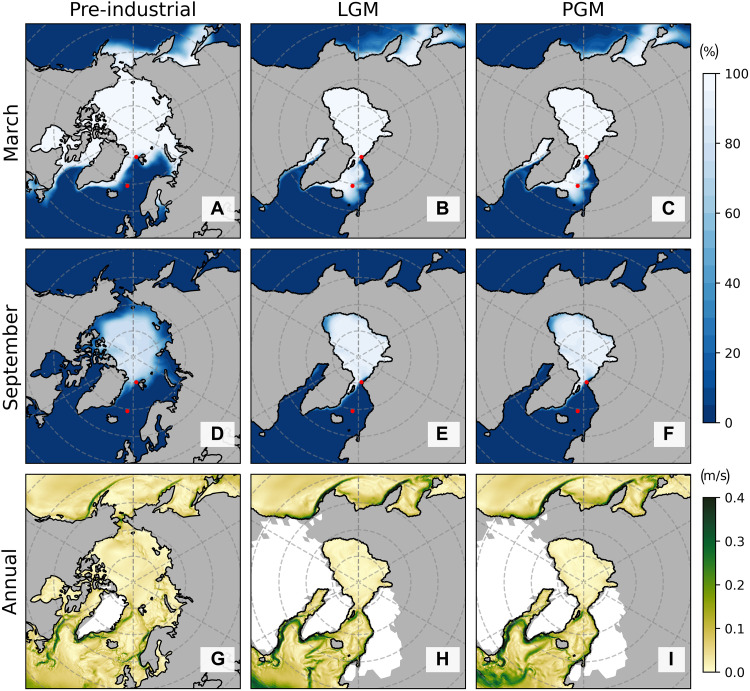
Simulated sea-ice concentration and surface current in pre-industrial, LGM and PGM states. Seasonal sea-ice concentration from earth system modeling in (**A** and **D**) PI, (**B** and **E**) LGM, and (**C** and **F**) PGM states. Red points denote sites 910A and MD99-2277. (**G** to **I**) Annual mean values of the sea surface current velocity amplitude.

Our proxy data reconstruction of marginal sea ice conditions in the AAG and Nordic Seas is supported by earth system modelling for preindustrial (PI) conditions, the LGM, and the Penultimate Glacial Maximum (PGM = MIS 6) (Fig. 3). The Alfred Wegener Institute–ESM (AWI-ESM2) is based on a multiscale modeling approach for paleoclimate questions (*45*, *46*), allowing for a high resolution in the dynamically relevant regions such as coasts, major upwelling regions, and high latitudes (see the fig. S4 for details). The PI and glacial setups follow the protocol of the Paleoclimate Modelling Intercomparison Project (PMIP) phase 4 (*47*) with prescribed land-sea mask and ice sheet configurations. The model simulations have been run for 1000 years into a quasi-equilibrium, and the mean over the past 100 years is shown (Fig. 3). Using the PI as representative for interglacial periods, our model simulations demonstrate year-round sea-ice coverage in the central Arctic Ocean (>80°N). Primarily ice-free conditions are simulated for most of the Nordic Seas due to the inflow of warm Atlantic water, while the East Greenland shelf remains sea-ice covered due to outflow of cold Arctic surface waters via the Transpolar Drift. In contrast, during two typical glacial periods (LGM and PGM; MIS 6), the model reveals year-round sea ice in the AAG and seasonal sea ice in the Nordic Seas (Fig. 3) with no clear indication of any interruption in ice drift during past glacial periods. The model output also implies the prevalence of the Transpolar Drift and Beaufort Gyre in the Arctic Ocean during extreme glacial states (i.e., the LGM and PGM) (Fig. 3). However, we emphasize that a truly coupled ice sheet/ice-shelf climate model remains limited at this point since either the climate (*30*), the ocean and ice (*48*), or the ice sheets (this study) are prescribed, something in need of attention for future modeling activities.

This alternating glacial-interglacial sea-ice scenario in the Nordic Seas thus mirrors our bioproductivity record from MD99-2277 (Fig. 2). During glacial periods, winter sea ice in the Nordic Seas would have resulted from subdued poleward heat transport from warm Atlantic water at the ocean-atmosphere interface (fig. S5). However, a simulated open-water corridor in the eastern Nordic Seas suggests a persistent influence of northward flowing, warm Atlantic waters, and strong katabatic winds originating from the adjacent ice masses in the Barents Sea and Scandinavia. Notably, the sea ice-open water conditions in the eastern AAG (Fig. 3) agree with surface water production of both sea-ice diatoms (i.e., IP_25_) and phytoplankton (epi-brassicasterol and dinosterol) at the 910A location during the LGM and PGM (Fig. 2). We note, however, that these open-water conditions in the eastern Nordic Seas are not consistent across different ESMs (fig. S5), possibly due to a highly uncertain Atlantic inflow into the AAG in the simulations. Our modeling results from the LGM and PGM simulations also further demonstrate that greenhouse gas concentration and orbital parameters play a minor role in controlling sea-ice conditions in the AAG and Nordic Seas. We note, however, that the sensitivity of ice sheet boundary conditions between the LGM and PGM (*49*) is not tested in this study (see the Supplementary Materials).

## DISCUSSION

From these proxy model simulation studies in the AAG and Nordic Seas, we conclude that, on suborbital to millennial timescales, persistent (year-round) to seasonal (autumn-late spring) sea-ice conditions prevailed during glacial times over the past 750,000 years. However, extremely low values for IP_25_ and phytoplankton biomarkers are seen in the AAG during the LGM and MIS 16, while for the AAG and Nordic Seas both IP_25_ and XRF Ca (clr) data show their absolute minima during MIS 16 (Fig. 2), pointing to particularly harsh sea-ice conditions in the AAG and Nordic Seas during these time periods. This implies that, for a short period (~1000 to 2000 years), MIS 16 may represent the only glacial interval where proxy evidence does not support the presence of open water or seasonal sea-ice conditions, raising the possibility—albeit speculative—of grounded or floating ice shelves in the region at that time. For the LGM, the spatial extent of the Eurasian ice sheets is very well constrained (*50*, *51*). While seasonal ice-free conditions are reported in the vicinity of the ice sheet front (*35*, *38*–*40*), perennial sea ice coverage occurred in the Eurasian sector of the Arctic during the LGM (*52*). Our data confirm that extensive sea ice cover limited bioproductivity and caused reduced sedimentation rates (<3 cm/1000 years) in the AAG. In contrast, the spatial extent of glacial ice in the Northern Hemisphere during MIS 16 is unknown, although is suggested to have been similar to the LGM (*51*). Glacial MIS 16 evolved from a gradual increase in ice volume that has characterized most full glacial periods since the MPT and marks the onset of ~100-ka glacial-interglacial variability (*53*). North Atlantic climate records indicate very cold bottom water temperatures and enhanced continental ice volume during MIS16 (*54*), which is consistent with limited bioproductivity inferred from our biomarker and XRF Ca (clr) records (Fig. 2). In support of these extreme glacial conditions during MIS 16, *p*CO_2_ concentrations fell to their lowest values during the Quaternary (<180 parts per million volume) for about 3000 years (Fig. 2) (*55*, *56*).

Together, our proxy data and model simulations suggest that a contiguous, pan-Arctic ice shelf did not exist in the AAG and Nordic Seas at virtually any time during past glaciations since the MPT. Exceptionally, we cannot rule out the possibility of short-lived and spatially restricted ice shelves during extreme glacial events (i.e., MIS 16) as indicated by geomorphic evidence on the Jan Mayen Ridge (~60 km west of MD99-2277) (*8*), perhaps during MIS 16 or older glaciations. Even so, the most compelling evidence from submarine landforms and boreholes for a possible grounding/floating ice shelf beyond the shelf edge in the AAG region is only evident for the MPT, at some time(s) between ~950,000 and 790,000 years ago (*19*, *32*). If a pan-Arctic ice shelf existed after this time, then it was most likely transient and regionally confined, leaving limited geomorphic or proxy signatures.

In this context, we note the need to distinguish between ice shelves and sea ice as distinct components of the cryosphere. Similarly, while our proxy resolution focuses primarily on orbital-scale changes, we acknowledge that suborbital variability (e.g., Heinrich events) may influence regional ocean-ice dynamics, especially in the Nordic Seas, where Atlantic Water inflow and complex stratification patterns could lead to strong spatial variability. These distinctions are important for interpreting the heterogeneity of past glacial Arctic climate and cryosphere configurations and can be further explored in future high-resolution proxy studies, combined with coupled climate ice sheet models, which have become recently available (*57*).

## MATERIALS AND METHODS

### Material

During ODP Leg 151, Hole 910A was drilled in 556.4-m water depth on the southern Yermak Plateau (80.2647°N, 6.59°E). In total, 34 m of almost undisturbed Pleistocene sediments was cored, and recovery was, on average, 71%, but 98% from 0 to 24.5 mbsf (*58*). In total, 32.5 m of sediments was recovered in piston core MD99-2277 from the eastern slope of the Iceland Plateau in the western Norwegian Sea (69.250°N, 6.3212°W) in 2800-m water depth by RV *Marion Dufresne* in 1999 (*59*). Nearby gravity core PS1243 (69.3718°N, 6.553°W) taken in 2711-m water depth was used for age correlation (*22*).

### Methods

#### Biomarker analyses

Following the methodology detailed in (*1*), biomarkers were extracted from freeze-dried subsamples (~5 g) from ODP Hole 910A. Before extraction, samples were spiked with internal standards 9-octylheptadec-8-ene (10 μl; 10 μg ml^−1^) and 5α-androstan-3β-ol, (0.1 μg) to permit quantification of HBIs and sterols, respectively. Samples were saponified in a methanolic KOH solution [~5 ml of H_2_O:MeOH (1:9) and 5% KOH] for 60 min (70°C). Hexane (3 × 2 ml) was added to the saponified content with supernatant solutions containing non-saponifiable lipids (NSLs), transferred with glass pipettes to clean vials, and dried over a gentle stream of N_2_ to remove traces of H_2_O/MeOH. NSLs were then resuspended in hexane (0.5 ml) and fractionated using column chromatography (SiO_2_; 0.5 g). Nonpolar lipids, including HBIs, were eluted with hexane (6 ml), and sterols were subsequently collected using 4:1 (v/v) hexane:methyl acetate (~7 ml). Following N_2_ blowdown (25°C), sterol-containing fractions were derivatised with *N*,*O*-bis(trimethylsilyl)trifluoroacetamide (100 μl; 70°C for 60 min). Each nonpolar fraction was further purified to remove saturated components using silver-ion chromatography (*60*) with saturated compounds eluted with hexane (2 ml) and unsaturated compounds, including HBIs, collected in a subsequent acetone fraction (3 ml). The analysis of HBI-containing fractions was carried out using gas chromatography–mass spectrometry (GC-MS) following the methods and operating conditions described prevously (*61*). Mass spectrometric analysis was carried out in total ion current (TIC) and selected ion monitoring (SIM) modes. The identification of HBIs and sterols was based on their characteristic GC retention indices and mass spectra (*16*, *18*, *62*). The quantification of all HBIs was achieved by comparison of mass spectral responses of selected ions (e.g., IP_25_, mass/charge ratio 350) in SIM mode with those of the internal standard and normalized according to their respective instrumental response factors, derived from solutions of known biomarker concentration, and sediment masses (*61*).

#### XRF core scanning

Avaatech XRD core scanning were used for inorganic geochemical analysis of MD99-2277 using a Rhodium x-ray source. The measurements were carried out in two runs at 20-mm steps with down-core and cross-core slits of 10 and 12 mm, respectively, using the following settings: (i) 10 kV, 1000 μA, 10-s counting time, no filter; and (ii) 30 kV, 2000 μA, 10-s counting time, Pd-thick filter. To overcome the subcompositional incoherence among variables, we calculated the centered log-ratio (clr) transformations by dividing each variable by the geometric means of its value and taking the logarithm (*63*). We use the XRF-Calcium (clr) record as an indicator of planktic foraminiferal production as outlined in the Supplementary Materials.

#### Chronology

ODP Hole 910A (80°15.882’N, 6°35.405′E, 556-m water depth) has a recovery rate of 98% for the upper 24.5 mbsf (*58*). Sediments are highly overconsolidated below ~19 mbsf, marked by large increases in bulk density and sediment strength (*58*). Initially, the upper boundary of the overconsolidated section is dated near the boundary between MIS 16 and MIS 17 (~0.66 Ma ago) (*64*). The stratigraphic framework for Hole 910A has later been revised by Knies, Matthiessen, Mackensen, Stein, Vogt, Frederichs, and Nam (*19*) proposing a hiatus (~0.79 to 0.95 Ma ago) at the top of the Matuyama Chron (~19.5 mbsf). This sequence, including the hiatus (~0.79 to 0.95 Ma ago), is attributed to a pronounced ice sheet advance or plowing of deep-drafted megabergs/remnants of an ice shelf (*19*, *28*, *29*, *32*, *64*). Sediments above the hiatus are marked by distinct color banding in the top 17 m of the sequence (*58*). Colors grade from brownish gray and dark olive gray to very dark gray on a 1- to 10-cm scale. Sediments in this sequence are predominantly composed of silty clays and clayey silts, and no further unconformities are reported (*58*).

Below, we outline analytical details and provide the age fix points for the stratigraphic framework. Briefly, the stable oxygen isotope data of *N. pachyderma* tests (125 to 250 μm) was generated at the AWI using a Finnigan MAT 251 isotope ratio gas mass spectrometer directly coupled to an automated carbonate preparation device (Kiel II) and calibrated via NIST19 international standard to the Vienna Peedee Belemnite (V-PDB) scale. The precision of the measurements at 1-σ based on repeated analyses of an internal laboratory standard (Solnhofen limestone) was better than ±0.08 for oxygen isotopes. For constructing a stable oxygen isotope stratigraphy of Hole 910A, the data published by Flower (*64*) and Knies *et al.* (*19*) were combined. Top and bottom of the studied interval (0.15 to 19.4 mbsf) are constrained by AMS^14^C ages and the Brunhes/Matuyama chron boundary (0.78 Ma ago). MIS events are identified and time-constrained through correlation with global δ^18^O stack (*24*). Further diagnostic biostratigraphic events for the upper Pleistocene include the highest occurrence of the calcareous nannofossil *Pseudoemiliania lacunosa* in late MIS 12 (~0.44 Ma ago) at 10.45 mbsf (*65*) and the maximum occurrence of the benthic foraminifera *Pullenia bulloides* in MIS substage 5.1 (0.08 Ma) at 1.95 mbsf.

The chronology of MD99-2277 has been published by Helmke, Bauch and Erlenkeuser (*20*, *21*). By correlating stable oxygen isotope data of *N. pachyderma*, carbonate content, IRD, and other sedimentological properties of the upper ~11 mbsf to well dated and nearby PS1243, the past five glacial-interglacial cycles covering the past ~0.44 Ma were identified by Bauch, Erlenkeuser, Helmke and Struck (*23*) and Helmke, Bauch and Erlenkeuser (*20*). The Brunhes/Matuyama chron boundary (0.78 Ma ago) is placed at ~18.84 mbsf (*20*, *21*). Between MIS 11 and the Brunhes/Matuyama chron boundary, continuous planktic δ^18^O data supported by carbonate content and IRD input, MIS events are identified and time-constrained through correlation with the global δ^18^O stack (*24*).

#### Calcium carbonate and planktic foraminifera

The bulk carbonate content [weight % (wt %)] in MD99-2277 and PS1243 was measured in the Holocene every 1 cm and for the rest, on average, every 5 cm, respectively, using a LECO C-200 carbon determinator at the GEOMAR Helmholtz Centre for Ocean Research, Kiel, Germany. For total carbon (TC) determination, subsamples of 300 to 400 mg were combusted at 1350°C, and the production of CO_2_ was determined. For total organic carbon (TOC) analysis, sub-samples of 400–450 mg were placed in carbon-free pervious ceramic combustion boats. These were placed on a heating plate at 50°C (± 5°C) and treated with 10 vol % hydrochloric acid (HCl) to remove inorganic carbon (carbonate) and subsequently rinsed with distilled water and dried in the drying oven prior to analysis. Calcium carbonate (CaCO_3_) content was computed via the following equations

IC(wt%)=TC(wt%)–TOC(wt%) (1)

$${\text{CaCO}}_{3}\;\left(\text{wt}\%\right)=\text{IC}\;\left(\text{wt}\%\right)\times 8.33$$ (2)

IC is the amount of inorganic carbon, and 8.33 corresponds to the stoichiometric calculation factor for further calculation of CaCO_3_. Results are given in wt %, and the SD of TC and TOC based on the repeated measurement of a standard was ±0.026 wt % (1σ, *n* = 8) and ± 0.028 wt % (1σ, *n* = 11), respectively.

The CaCO_3_ content in PS1243 corresponds to the variable production of planktic foraminifera. Planktic foraminifera in PS1243 were studied in the >125-μm size fraction and are expressed as specimens per gram of dry bulk sediment (No./g Sed). Cross correlation between CaCO_3_ and number of planktic foraminifera reveal a significant coefficient of correlation (*R* = 0.87; *n* = 83) and *P* value (<0.001). The carbonate content (wt.%) in both records, PS1243 and MD99–2277, coherently varies despite different sampling resolution. In fig. S2, we illustrate this further for the time period between ~440,000 and ~370,000 years ago (late MIS 12 to early MIS 10). The CaCO_3_ content is well reflected in the XRF-Calcium (clr) record with a high correlation coefficient (*R* = 0.87; *P* < 0.001, *n* = 377) (fig. S3). This significant correlation allows the inference of using XRF-Calcium (clr) as an indicator of variable surface water production of planktic foraminifera at site MD99-2277 location.

#### Earth System Model

The AWI-ESM (AWI-ESM2) is an extension of AWI-CM2 (*66*) for earth system modeling. It consists of the ocean-sea ice component FESOM2 (*67*), the atmospheric component ECHAM6 (*68*), and the land component JSBACH (*69*, *70*) with dynamic vegetation and a hydrological discharge submodel calculating global surface runoff. The atmospheric and land components ECHAM6/JSBACH apply a T63 Gaussian grid in the horizontal. ECHAM6 applies 47 vertical layers, and JSBACH applies five soil layers with 11 vegetation types. The ocean component FESOM2 applies unstructured meshes, which makes it feasible to resolve coastal, tropical, and polar areas with a relatively high resolution by a moderate computational effort. The mesh resolution at the Arctic-Nordic Seas is around 25 km (fig. S4). In the vertical, FESOM2 applies 47 progressively thicken layers within a *z*-star coordinate. We do not apply interactive ice sheets in the model. Therefore, the ice sheet in our ESM is prescribed and constant during the simulations. The ESM configuration follows previous modeling studies and has been validated for different climate states (*71*, *72*).

For this study, we designed three experiments for different climate stages, namely, PI, LGM and PGM. The simulations represent one interglacial period and two glacial periods. Considering that ice sheet reconstruction contains larger uncertainty for PGM than LGM state, for both LGM and PGM simulations, the ice sheet orography and land-sea mask applied correspond to the LGM state (21 ka ago) from the GLAC-1d database (*73*). The differences between our LGM and PGM simulations are reflected in the greenhouse gas concentration and orbital parameters (*47*, *74*). The reason for LGM/PGM configuration consists of three aspects: (i) LGM and PGM are characterized by similar global total ice sheet volume and global mean sea level (~120 m lower than today); (ii) Ice-sheet reconstruction for the PGM state contains larger uncertainties than LGM state; (iii) controlling the ice-sheet configuration between LGM and PGM helps to examine the role of orbital configuration in altering the two glacial periods (*49*). All experiments have been spun up for over 1000 years. The results we show are averaged from the past 100-year simulation.

In addition, we compare our LGM simulation with other models from the Paleoclimate Modeling Intercomparison Project (PMIP). According to our main focus in this study, we demonstrate a comparison of March sea-ice concentration from different LGM simulations (fig. S5). ESMs from PMIP3/PMIP4 show large disagreements in the sea-ice condition in the Nordic Seas. Moreover, AWI-ESM2 is one of the ESMs that supports an ice-free condition at the eastern coast of the Nordic Seas in boreal winter.
