# Supplementary material for: Seasonal sea ice characterized the glacial Arctic-Atlantic gateway over the past 750,000 years
Source: Sci Adv. 2025 Jul 4;11(27):eadu7681. doi: 10.1126/sciadv.adu7681 (PMC12227056; doi:10.1126/sciadv.adu7681)
Supplement: Supplementary file 1 — Figs. S1 to S5 Tables S1 to S4 [file sciadv.adu7681_sm.pdf]

Science Advances
AAAS

Supplementary Materials for

Seasonal sea ice characterized the glacial Arctic-Atlantic gateway over the past 750,000 years

Jochen Knies *et al.*

Corresponding author: Jochen Knies, jochen.knies@ngu.no

Sci. Adv. **11**, eadu7681 (2025)
DOI: 10.1126/sciadv.adu7681

This PDF file includes:

Figs. S1 to S5
Tables S1 to S4

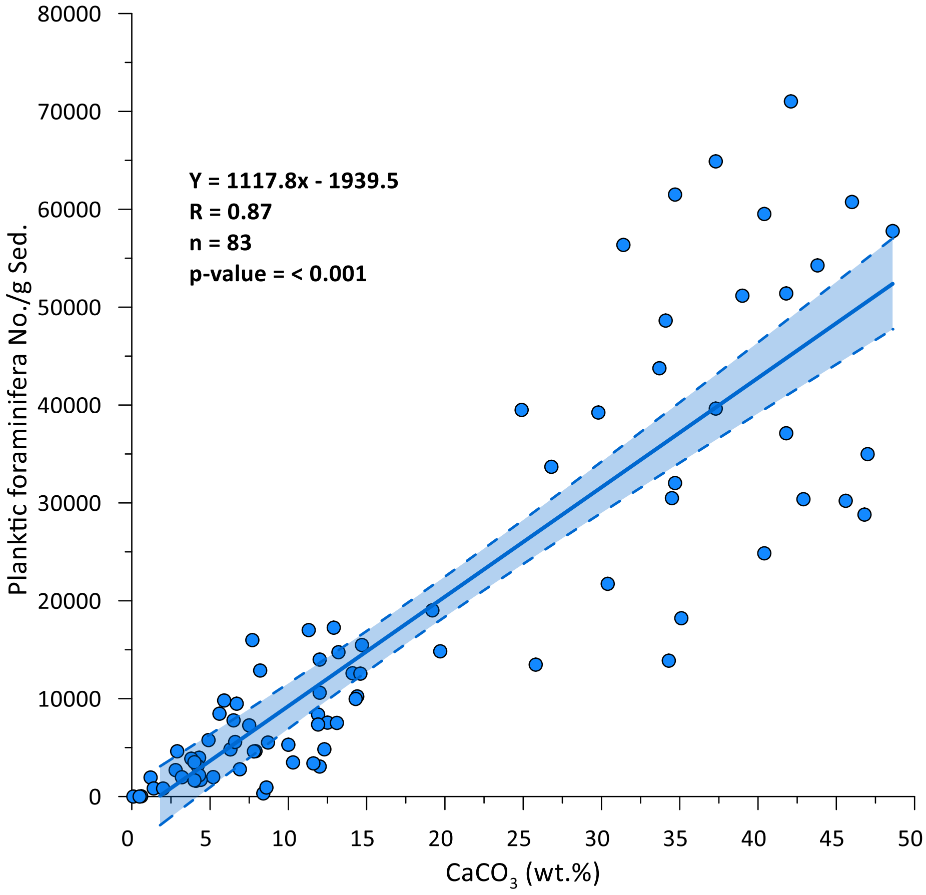

Figure S1: Cross plot between planktic foraminifera (No./g Sed) and calcium carbonate ($CaCO_3$) (wt. %) in PS1243. See core location of PS1243 in Fig 1 of the main text. Note with correlation coefficient R = 0.87 ($p<0.001$, n=83) and 95% confidence intervals (light blue bars).

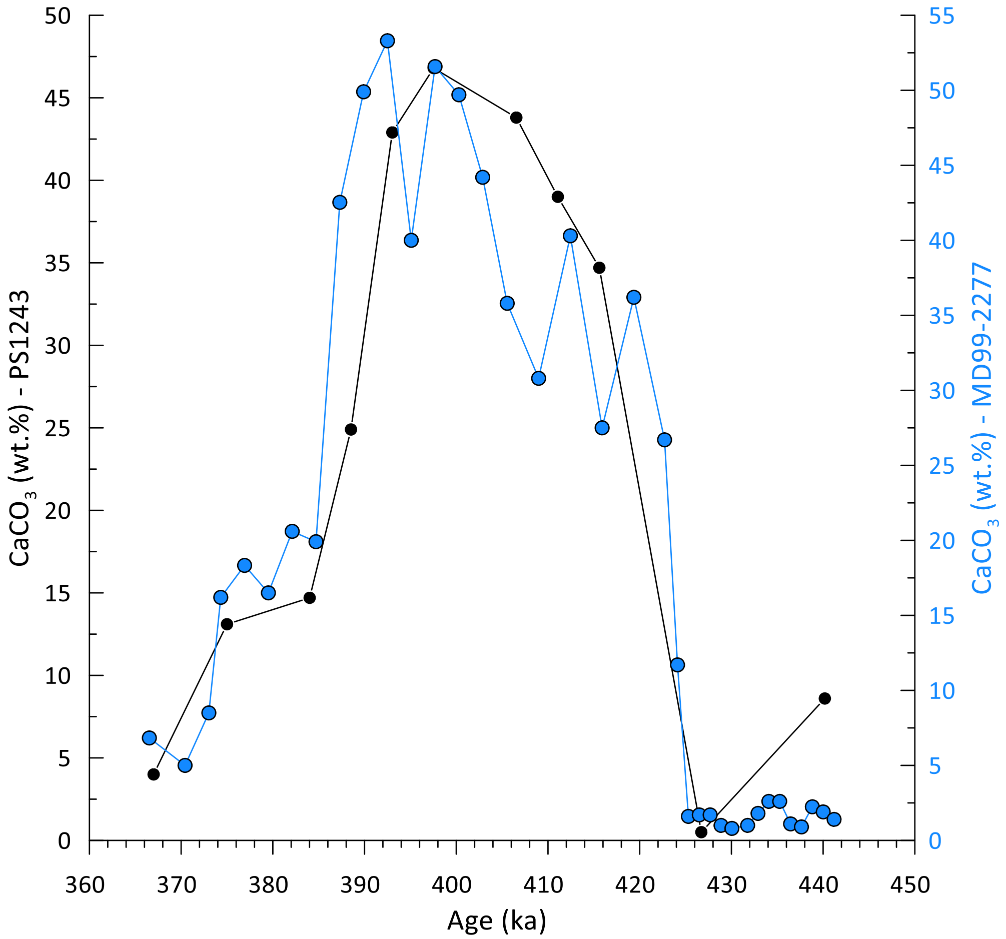

Figure S2: Calcium carbonate ($CaCO_3$) content (wt. %) in PS1243 (black) and MD99-2277 (blue) between ~440 000 (late MIS 12) and 370 000 years (early MIS 10). Both cores were spliced together on the basis of the matching $CaCO_3$ content across MIS 11.

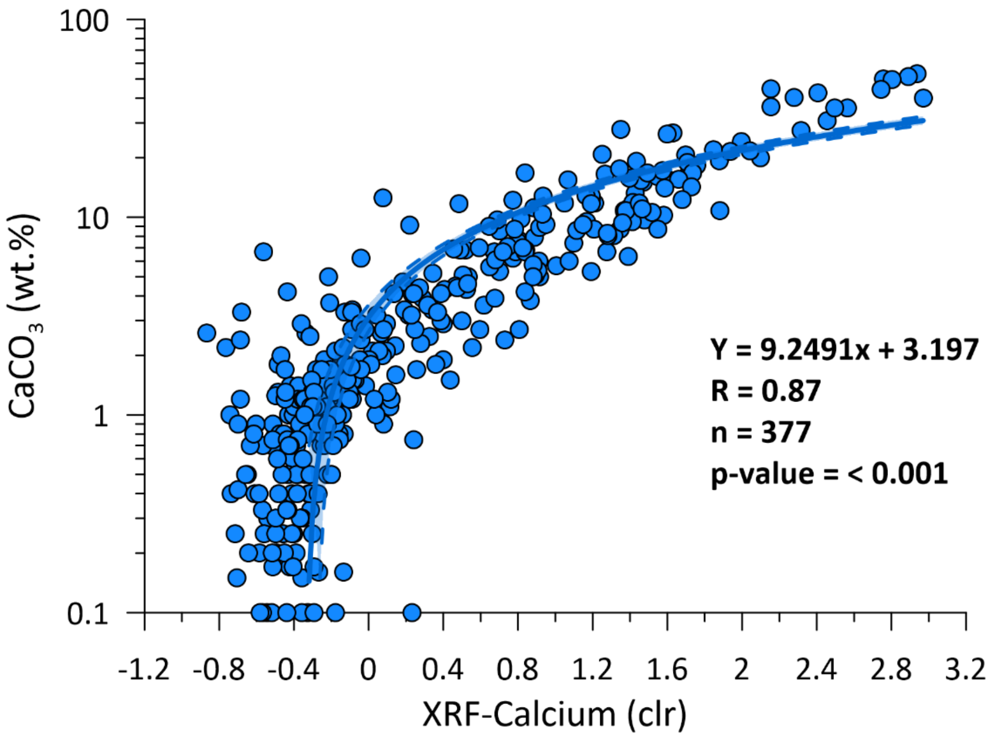

Figure S3: Cross plot between calcium carbonate ($CaCO_3$) (wt. %) and XRF-Calcium (clr) in MD99-2277. Note the correlation coefficient R = 0.87 ($p<0.001$, n=377) and 95% confidence intervals (light blue bars).

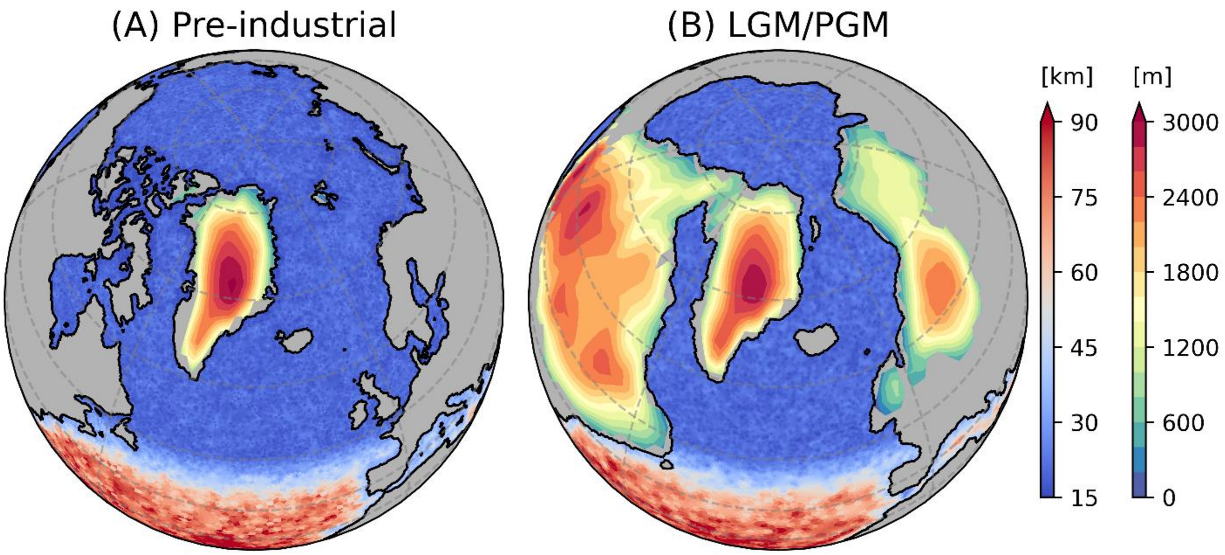

Figure S4: Model meshes for pre-industrial, LGM and PGM simulations. Colours in land areas show ice-sheet orography. Colours in ocean areas show ocean mesh resolution. The LGM mesh is applied in both LGM and PGM simulations.

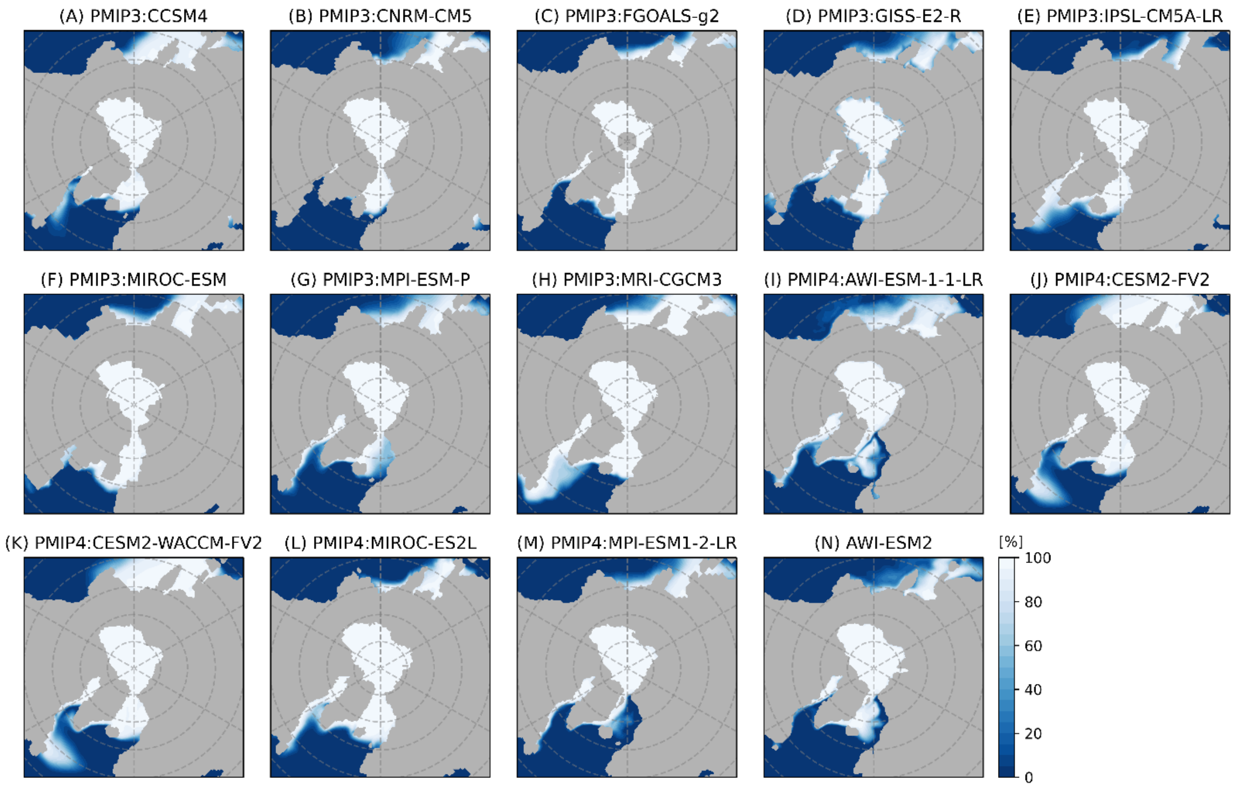

Figure S5: A model intercomparison of March sea-ice concentration at the LGM state.

Panels (A–H) represent model results from PMIP3, and panels (I–M) represent model results from PMIP4. Panel (N) shows results from AWI-ESM2.

[CMIP data set], https://aims2.llnl.gov, last access: 02 February 2025.

Table S1. ODP Hole 910A age model. ODP Hole 910 age model, fixpoints, and sedimentation rates.

Age fixpoints	Depth cmbsf	Age (ka)	Principal Reference	Age assignment	LSR cm/kyr
AMS ^{14}C	34.5	21.4	17		
MIS 2.2	35	20	17	22	2.7
MIS 4.2	147	62	17	22	2.5
MO P. bulloides	195	81	17	22	4.6
MIS 6.0	420	130	17	22	3.0
MIS 6.2	450	140	17	22	3.1
MIS 6.6	590	185	17	22	1.9
MIS 8.4	750	270	17	22	1.2
MIS 10.2	835	342	17	22	1.5
MIS 12.0	960	424	17	22	8.0
MIS 12.2	1040	434	17	22	0.8
HO P. lacunosa	1045	440	62	22	3.8
MIS 13.2	1320	513	17	22	3.8
MIS 14.2	1414	538	17	22	9.6
MIS 14.4	1510	548	17	22	2.7
MIS 14/15	1550	563	17	22	1.6
MIS 15/16	1645	621	17	22	0.7
MIS 16/17	1685	676	17	22	3.9
MIS 18.2	1848	718	17	22	1.5
B/M boundary	1945	781	17	23	

LSR = Linear Sedimentation Rate
MIS = Marine Isotope Stage
P. bulloides = Pullenia bulloides
P. lacunosa = Pseudoemiliani lacunosa
MO = Maximum Occurrence, HO = Highest Occurrence
B/M = Brunhes/Matuyama

Table S2. Stable oxygen isotopes of Hole 910A. Source: https://figshare.com/articles/dataset/ODP_910A_stable_oxygen_isotopes/21506571?file=38112678 .

Table S3. HBI and sterol biomarker data of 910A. Source: https://figshare.com/articles/dataset/ODP_910A_biomarker_data/21506478?file=38112657 .

Table S4. XRF calcium (clr) in MD-992277. Source: https://figshare.com/articles/dataset/X-ray_fluorescence_scanning_calcium_centre-log_ratio_transformation_clr_data_in_sediment_core_MD99-2277/26968624?file=49077232 .
